# Arthroscopic Rotator Cuff Repair With Patch Augmentation Using an Acellular Dermal Matrix Allograft Into the Triple Row‐Suture Bridge Technique

**DOI:** 10.1002/atn2.70042

**Published:** 2026-05-07

**Authors:** Woo‐Yong Lee, Hyung‐Jin Chung, Kyung‐Cheon Kim, Jae‐Young Park

**Affiliations:** ^1^ Department of Orthopedic Surgery Chungnam National University Hospital Chungnam National University College of Medicine Daejeon Republic of Korea; ^2^ Department of Orthopedic Surgery Chungnam National University Sejoing Hospital Chungnam National University College of Medicine Sejong Republic of Korea; ^3^ Department of Orthopedic Surgery Tantan Hospital Daejeon Republic of Korea; ^4^ Department of Orthopedic Surgery Daejeon Eulji University Medical Center Eulji University School of Medicine Daejeon Republic of Korea

## Abstract

Patch augmentation with acellular dermal matrix (ADM) allograft is increasingly recognized as an effective method to enhance healing rates following rotator cuff repair in cases of massive rotator cuff tears. However, there is still variability in how patch augmentation is performed. In this study, the authors present a patch augmentation using an ADM allograft into the triple row‐suture bridge technique for treatment of large to massive rotator cuff tear. This method, characterized by knotting the medial row sutures over the ADM allograft, is designed to enhance healing by increasing the contact area among the graft, the rotator cuff tendon, and the footprint, while reducing direct pressure on the tendon.

## Graphical abstract

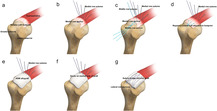

**VIDEO 1:** The video of repair technique of patch augmentation using an acellular dermal matrix allograft into the triple row‐suture bridge technique. Video content can be viewed at https://doi.org/10.1002/atn2.70042.

The primary objective of rotator cuff repair is to facilitate tendon‐to‐bone healing within the anatomical footprint of the rotator cuff tendon.^1^ However, this goal is often difficult to achieve in cases of large to massive rotator cuff tears due to the inability to adequately reduce the retracted tendon to its footprint or the excessive tension required to do so. As a result, the management of massive rotator cuff tears remains a significant challenge for orthopaedic surgeons. The prevalence of massive tears has been reported to range from 10% to 40% of all rotator cuff tears.^2^

Since the introduction of acellular dermal matrix (ADM) allograft, patch augmentation using this material has been widely adopted for patients with large to massive rotator cuff tears and poor tissue quality. Patch augmentation with ADM allograft is increasingly recognized as a method to enhance the healing rates in massive rotator cuff tears.^3^, ^4^ This technique provides enhanced structural integrity and biomechanical load to failure.^5^, ^6^ It is particularly indicated in patients with large to massive rotator cuff tears, retears, or chronic tears associated with poorer tissue quality.^7^

In this article, we describe a rotator cuff repair procedure using patch augmentation with an ADM allograft, based on the triple row‐suture bridge technique. This method addresses key technical considerations, including graft preparation, passage, and placement, as well as suture management and the role of the assistant.

## SURGICAL TECHNIQUE

The surgical technique is shown in Video 1.

### Positioning and Preparation

The patient is placed in the beach‐chair position under general anesthesia combined with an interscalene nerve block. After sterile draping, the incision sites are marked, and the surgical plan is confirmed.

### Arthroscopy Portals

Diagnostic arthroscopy of the glenohumeral joint is performed through a standard posterior viewing portal. The transition to the subacromial space and lateral portal is established as the working portal. Acromioplasty is performed in patients with subacromial osteophytes. After switching the viewing portal to the lateral portal, an anterolateral portal is created and fitted with an 8‐mm cannula (PassPort, Arthrex) to serve as the working portal for the rotator cuff repair procedure.

### Placement of Suture Anchors on the Greater Tuberosity

Following bursectomy and debridement of the degenerative tissues at the rotator cuff tear site, the remaining rotator cuff tendon and surrounding tissues are thoroughly released to ensure adequate tendon excursion. Using a grasper, we assess whether the torn tendon can be fully reduced and whether patch augmentation is feasible. If the mobilized tendons fail to completely cover the rotator cuff footprint, medialization of the footprint by approximately 5 mm is performed by resecting the lateral margin of the articular cartilage.

**FIGURE 1:**
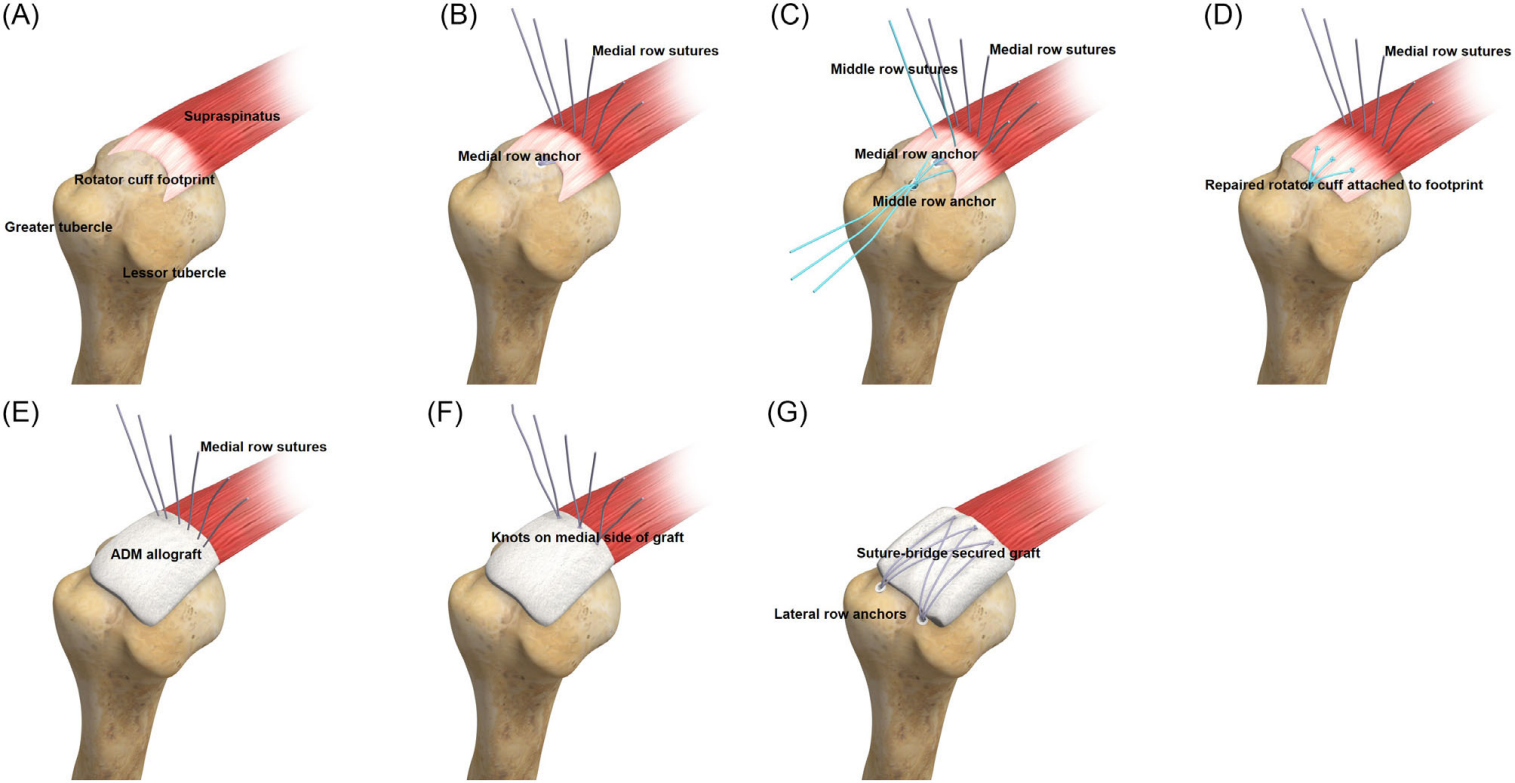
Schematic illustration of the right shoulder in the beach‐chair position. The scheme of repair technique of patch augmentation using an acellular dermal matrix allograft into the triple row‐suture bridge technique. (A) Preparation of the torn rotator cuff and the footprint. (B) A suture anchor is inserted into the medial row, and sutures are passed through the muscle‐tendon junction. (C) A suture anchor is inserted into the middle row, and sutures are passed approximately 5 mm lateral to the sutures from the medial row anchor. (D) The sutures from the middle row anchor are used for simple sutures to reduce the torn tendon. (E) The sutures from the medial anchor were sequentially retrieved and passed through the medial aspect of the graft in order. (F) The sutures were knotted on the medial side of the graft. (G) Graft is fixed to the humerus using 2 lateral anchors by suture bridge technique.

**FIGURE 2:**
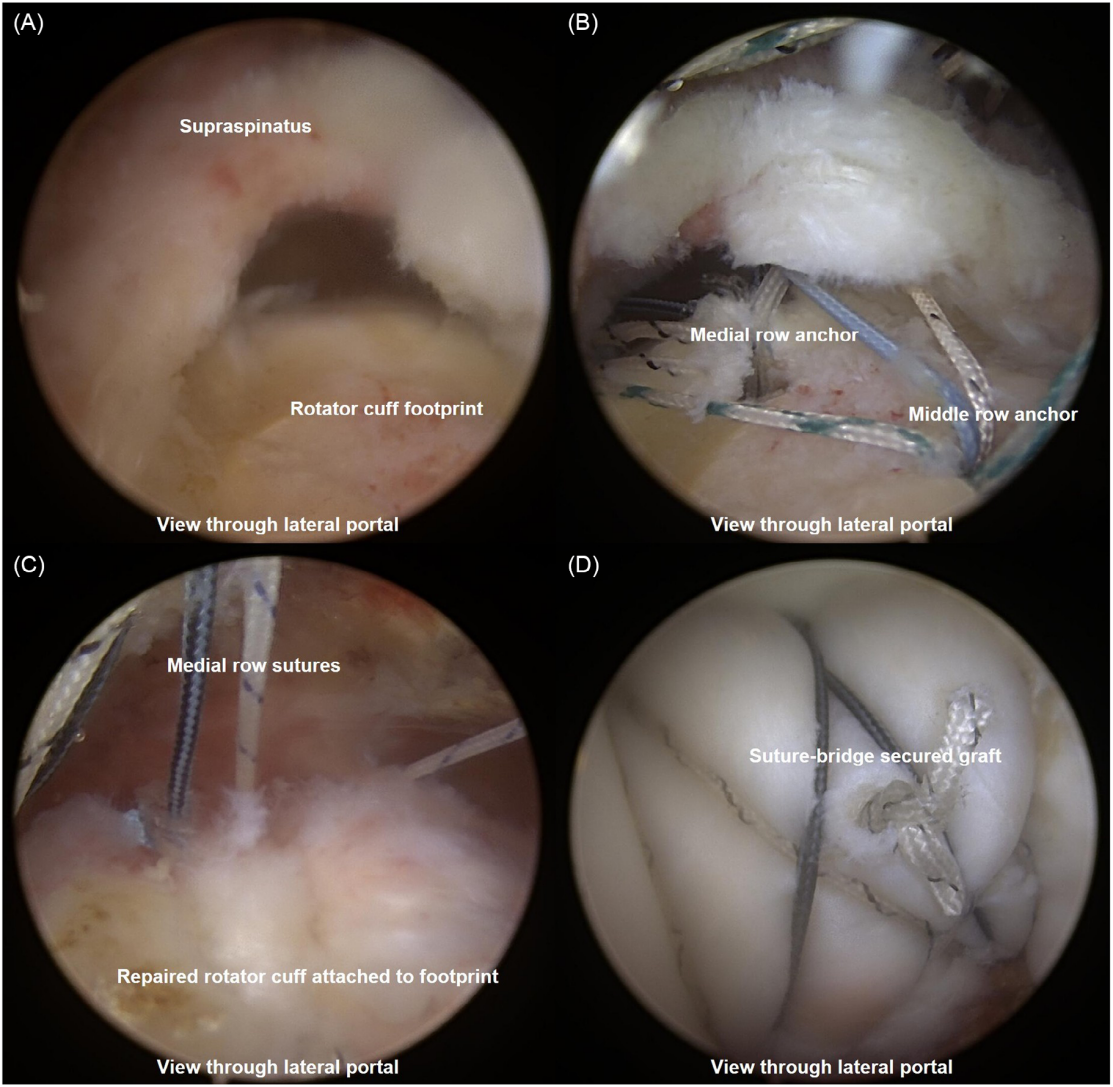
Schematic illustration of the right shoulder in the beach‐chair position. A 69‐year‐old woman has a full thickness supraspinatus tear. (A) Preparation of the torn rotator cuff and the footprint. (B) Suture anchors are inserted into the medial and middle rows, followed by suture passing to rotator cuff. (C) The sutures from the middle row anchor are used for simple sutures to reduce the torn tendon. (D) The acellular dermal matrix allograft is secured using the suture bridge technique.

The greater tuberosity is completely exposed, and a shaver and burr are used to decorticate the bone bed to facilitate graft‐to‐bone healing (Figures 1A and 2A). A superolateral portal is established via additional small incision at the anterolateral border of the acromion, and suture anchors are inserted into the greater tuberosity. Triple‐loaded anchors are used for the medial row and placed just lateral to the cartilage adjacent to the footprint. Depending on the tear size, 1 or 2 medial anchors are inserted. The sutures from the medial row anchors are placed at the muscle‐tendon junction, approximately 12‐15 mm medial to the lateral edge of the torn tendon (Figure 1B). Knot‐tying on the rotator cuff is deferred at this stage and sutures are brought out through the superolateral portal.

For the middle row, triple‐loaded anchors are also used and placed at the lateral edge of the footprint. One or 2 anchors are inserted depending on the tear size, and sutures from these anchors are placed approximately 5 mm lateral to those from the medial row (Figures 1C and 2B). Knot‐tying of the middle row anchors is performed using a simple suture technique, similar to the single‐row repair method, to reduce the torn tendon (Figures 1D and 2C).

### Graft Preparation and Extracorporeal Suture Management

**FIGURE 3:**
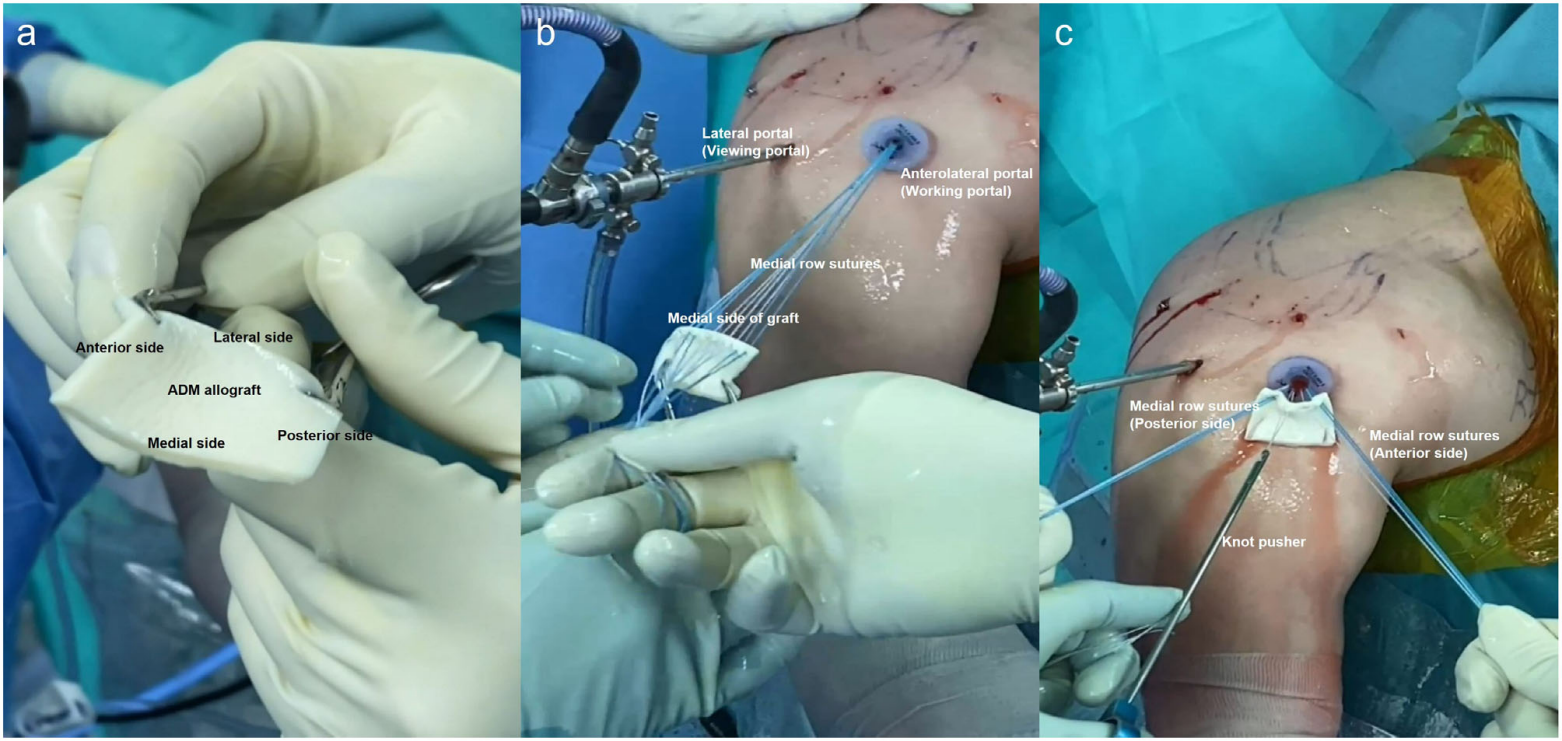
Schematic illustration of the right shoulder in the beach‐chair position. (A) The acellular dermal matrix (ADM) patch was positioned transversely, with its anterior and posterior edges grasped using mosquito forceps. (B) The sutures from the medial anchor were sequentially passed through the medial margin of the ADM patch. (C) The free ends of anterior and posterior suture pairs were simultaneously pulled by the assistant, and the 2 middle sutures were loaded into a knot pusher.

The ADM allograft is tailored to match the tear size, and placed transversely, with the tendon side of the graft (reticular side of the patch) facing downward. The anterior and posterior edges of the graft are held with 2 mosquito forceps by the assistant (Figure 3A). The sutures from the medial anchor are sequentially retrieved through the cannula from anterior to posterior and passed through the medial aspect of the graft in order (Figure 3B).

### Graft Insertion Into the Subacromial Space and Patch Augmentation

**FIGURE 4:**
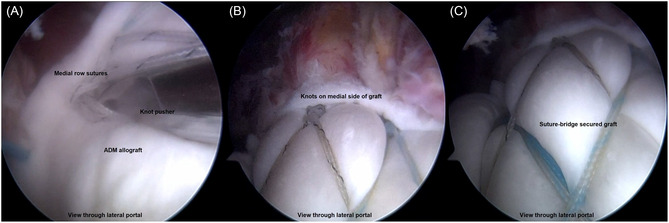
Schematic illustration of the right shoulder in the beach‐chair position. (A) The sutures from the medial anchors were tied on the medial side of the graft. (B) Knots on the medial side of the graft. (C) Graft was secured to the humerus using 2 lateral anchors.

The free ends of anterior and posterior suture pairs are simultaneously pulled by the assistant, and the 2 middle sutures are loaded into a knot pusher (Arthrex) (Figure 3C) to advance the graft into the subacromial space through the cannula of the anterolateral portal (Figure 1E). After the graft is inserted into the subacromial space, an arthroscopic examination is performed to check for suture tangling or loose strands. The sutures from the medial anchors are then tied on the medial side of the graft (Figures 1F and 4A). Finally, the graft is secured to the humerus using 2 lateral anchors via the suture bridge technique (Figures 1G, 2D, and 4B,C). Pearls and pitfalls are listed in Table 1.

**TABLE 1:** Pearls and Pitfalls

Pearls
Perform thorough bursectomy and soft tissue release
Maintain constant tension on the sutures during handling
Perform the extracorporeal procedure quickly and efficiently
When inserting the graft into the subacromial space, the remaining sutures (not loaded into the knot pusher) should be pulled simultaneously

Pitfalls
Inadequate bursectomy or soft tissue release may obscure the arthroscopic field
Improper suture handling may cause suture entanglement
Delay in the extracorporeal procedure may increase the risk of infection

## DISCUSSION

Patch augmentation using ADM allograft has emerged as a widely accepted and validated technique for treating large to massive rotator cuff tears.^8^ The ADM allograft is processed to become acellular, thereby reducing immunogenicity while preserving the integrity of the collagen extracellular matrix to provide mechanical strength and serve as a scaffold for new host tissue regeneration.^9^ Numerous previous studies have reported that patch augmentation results in superior healing rates compared with repairs without augmentation in cases of large rotator cuff tears.^10^

However, there is still variability in how patch augmentation is performed, with variations often depending on individual surgeon preferences. The technique described in this article is based on a triple row‐suture bridge technique with patch augmentation, emphasizing the importance of tying the medial row sutures over the ADM allograft. In current practice, medial row sutures are often either tied or left untied before placing the allograft on top, followed by lateral row fixation. This conventional method may lead to insufficient medial compression of the ADM allograft.

In contrast, the technique shown in this article involves tying the medial row sutures over the graft, which is expected to enhance medial compression of both the graft and the underlying rotator cuff tendon. This approach may increase the contact area and contact pressure between the ADM allograft, the repaired tendon, and the footprint, thereby promoting a stronger biological and mechanical environment for healing and enabling watertight structural integrity.

Moreover, tying sutures over the graft rather than directly on the tendon may help prevent suture cut‐through and reduce hypoxic damage to the rotator cuff, potentially lowering the incidence of type II retears. Another advantage of this technique is its compatibility with other procedures commonly performed for massive rotator cuff tears. For example, if sufficient reduction cannot be achieved despite adequate release and footprint medialization, muscle advancement can be employed to improve tendon mobility prior to graft application.

Additionally, in cases where the tendinous portion is severely deficient and full footprint coverage is unachievable, the ADM allograft may serve as a biologic tuberoplasty, providing a mechanical buffer effect.

There are several considerations regarding the technique introduced in this study. Even when medial row knots are tied over the patch, additional pressure may be exerted at that site, which could potentially increase the risk of type II retears. In addition, the use of multiple anchors may cause crowding of the sutures, leading to entanglement if appropriate handling by an assistant is not provided. Skilled assistance is also required during extracorporeal graft handling, and the extracorporeal procedure itself may potentially increase the incidence of infection. Further considerations are listed in Table 2.

**TABLE 2:** Advantages and Disadvantages

Advantages
Enhance medial compression
Increase the contact area and contact pressure between the ADM allograft and the repaired tendon
Pevent suture cut‐through and reduce hypoxic damage
Compatible with other procedures
Provides a biologic tuberoplasty effect (mechanical buffer)

Disadvantages
Additional pressure due to medial row knots
Crowding of the sutures, leading to potential entanglement
Skilled assistance is required
The extracorporeal procedure may potentially increase the incidence of infection

ADM, acellular dermal matrix.

Nevertheless, the patch augmentation technique introduced in this study may serve as a standardized and reproducible treatment option for large to massive rotator cuff tears. Our method, which incorporates systematic portal placement and a sequential knot‐tying strategy, does not require specialized instruments. Given its relative simplicity and short learning curve, this technique may help reduce operative time and improve surgical efficiency.

## DISCLOSURES

The authors (W‐Y.L., H‐J.C., K‐C.K., J‐Y.P.) declare that they have no known competing financial interests or personal relationships that could have appeared to influence the work reported in this paper.
